# 8.M. Round table: Health literacy and vaccine literacy as determinants of vaccine acceptance: a critical discussion

**DOI:** 10.1093/eurpub/ckac129.517

**Published:** 2022-10-25

**Authors:** Organised by: EUPHA Working Group Health Literacy, EUPHA-HP, Technical University Munich, Public Health Austria, Careum Switzerland, Global Health Literacy Academy, Chair persons: Orkan Okan (EUPHA-HP), Kristine Sørensen (Denmark)

## Abstract

In 2019, Sars-Cov-2 caused the greatest pandemic the modern world has ever faced. The pandemic was unprecedented regarding its effects on all aspects of society. Since the outbreak, debates on vaccines have been elevated in public health. The pandemic also emerged into a game changer regarding health communication and information delivery. With digital communication technologies, the Internet and Social Media being the most important tools to discuss health matters, exchange health knowledge and get advice from peers, every human has been part of a global communication network discussing the pandemic, related policies and vaccines. The digital realm allowed everybody to contribute to the state of Covid-19 related health information and absorb them. Altogether, this led to an overabundance of accurate and false information circulating the digital world, which culminated into the information epidemic (infodemic). Early on in the pandemic, it became obvious that people need competencies enabling them to navigate digital information environments, manage (digital) health information and to use digital health services that were accelerated through Covid-19. While health literacy and vaccine literacy were undervalued at the time, policy makers and practitioners soon highlighted their critical role in mitigating the spread of coronavirus, for protection against infection and increasing adherence to public health emergency measures. In context of the Covid-19 pandemic, health literacy enables people to find, understand and critically appraise relevant information and use it for prevention behaviour. Since it empowers individuals to mitigate the effects of the pandemic, health literacy is seen as a social vaccines. Vaccine literacy is a sub-dimension about health literacy, which especially became important when global roll-out of Covid-19 vaccines began in 2021. Vaccine literacy helps people to understand what a vaccine is, why it is relevant to get vaccinated and how it protects oneself and others. In addition, vaccine literacy empowers people to find vaccine-related information and judge about vaccine claims. The purpose of this roundtable is to discuss research findings on health literacy and vaccine literacy in relation to Covid-19, the determinants of vaccine acceptance, vaccine hesitancy and vaccine attitudes, generated in different European studies: (1) the international trend study HLS-Covid-19 conducted in Germany, Austria and Switzerland, (ii) HLCA-Kids-NRW on coronavirus-specific health literacy in primary schoolchildren (Germany) and (iii) the HLS19 European Health Literacy Survey. While the roundtable aims at introducing empirical findings, each panelist will provide a statement related to the roundtable theme based on the findings of their study. Together with the audiences, we will discuss about lessons learned from the pandemic and how to utilize health and vaccine literacy to increase vaccine acceptance.

**Key messages:**

• Health literacy is a social vaccine and empowers people to manage health information and increase vaccine acceptance.

• Vaccine literacy is a sub-dimension of health literacy and in particular is useful when addressed as part of public health emergency strategies.

**Speakers/Panellists:**

**Orkan Okan**

Technical University Munich, Munich, Germany

**Kristine Sørensen**

Global Health Literacy Academy, Risskov, Denmark

**Robert Griebler**

Austrian National Public Health Institute, Vienna, Austria

**Saskia De Gani**

Careum Center for Health Literacy, Zürich, Switzerland

**Torsten Michael Bollweg**

Technical University Munich, Munich, Germany

